# A comparative study of spinal cord compression management in metastatic prostate cancer: Teaching versus non‐teaching hospitals in the United States

**DOI:** 10.1002/cam4.6845

**Published:** 2023-12-26

**Authors:** Omid Yazdanpanah, Aditya Mahadevan, Aditi Sharma, David J. Benjamin, Arash Rezazadeh Kalebasty

**Affiliations:** ^1^ Division of Hematology and Oncology, Internal Medicine Department University of California Irvine Orange California USA; ^2^ Division of Hematology/Oncology Wayne State University School of Medicine/Karmanos Cancer Institute Detroit Michigan USA; ^3^ Hoag Family Cancer Institute Newport Beach California USA

**Keywords:** non‐teaching hospital, prostate cancer, radiation, spinal cord compression, surgery, teaching hospital

## Abstract

**Background:**

Spinal cord compression (SCC) in metastatic prostate cancer (MPC) is a critical complication and multiple factors influence the optimal therapeutic strategy. We investigated the differences in practice patterns between teaching hospitals (TH) and non‐teaching hospitals (NTH) across the United States.

**Method:**

Using the National Inpatient Sample Database (NIS), we performed a retrospective study on hospitalizations with MPC and SCC between 2016 and 2020 in US. We compared demographic factors, comorbidities, treatment modalities, duration of hospitalization, financial expenditures, and mortality between TH and NTH. We also examined the patients' characteristics and outcomes in TH and NTH based on their chosen therapeutic strategy.

**Results:**

We identified 11,380 admissions with metastatic prostate cancer and SCC; 9610 in TH and 1770 in NTH. The median cost of hospitalization was $21,922 in TH and $15,141 in NTH. Although the median age and Charlson comorbidity score did not differ between two groups, patients in TH were more likely to receive intervention (radiation or surgery) compared to NTH (Surgery: 28.2% in TH vs. 23.0% in NTH & Radiation: 12.1% in TH vs. 8.2% in NTH). Mortality was lower in TH than NTH (4.5% vs. 7.9%). In both TH and NTH, a higher proportion of patients with private insurance underwent surgery (TH: Surgery 25.1% vs. Radiation 18.8% & NTH: Surgery 27.0% vs. 6.9%). Black patients were more likely to receive radiation than surgery in TH (34.2% vs. 26.8%).

**Conclusion:**

This study showed a greater percentage of patients underwent surgical intervention at TH compared to NTH. Additionally, the type of insurance and racial background were associated with distinctive treatment approaches.

## 1 INTRODUCTION

Prostate cancer composes a significant proportion of diagnosed cancers worldwide, with an incidence of over 288,000 cases annually in the United States.^1^ Risk factors include age, ethnicity, family history, as well as lifestyle factors including red meat consumption.^2^, ^3^, ^4^ The management of prostate cancer incurs significant costs, often exceeding $55,000 per person‐year in patients with metastatic disease.^5^

Bone metastases are a common complication of patient with prostate cancer, occurring in over 90% of patients over disease course.^6^ Bone metastases are detected through a variety of imaging techniques including MRI, CT, and PET scans.^7^, ^8^ In patients with bone metastases, complications are often collectively referred to as skeletal‐related events (SRE), which include pathologic fractures, the need for radiation or surgery, or spinal cord compression.^9^ The most feared SRE is cord compression, occurring in approximately 9% of admissions related to skeletal‐related‐events.^10^ For patients suffering from cord compression, admission costs often exceed $60,000, adding to the significant cost per year in patients with metastatic prostate cancer.^10^

Management of spinal cord compression is complex and often involves a combination of high‐dose steroids, radiation, and surgical decompression.^11^ In unstable patients who are not good surgical candidates, interventional radiology procedures may have additional utility.^12^ While steroids are always indicated, the choice between radiation and surgical intervention depends on several factors, including tumor radiosensitivity, spinal instability, and medical comorbidities.^11^, ^13^, ^14^ When possible, combining surgical decompression with radiation therapy may yield superior clinical outcomes.^15^ Despite well‐established evidence for the management of cord compression, management of spinal cord compression varies based on patient's clinical status, race and insurance status.^16^, ^17^, ^18^, ^19^

Despite the existence of strong, evidence‐based management of malignant spinal cord compression in prostate cancer patients, hospital‐level differences exist in prostate cancer quality‐of‐care.^20^ In the current literature, there is a lack understanding of differences in practice patterns between academic and non‐academic hospitals, as well as the factors that influence these differences. This study's objective was to examine differences in management of spinal cord compression in patients with metastatic prostate cancer between teaching hospitals (TH) and non‐teaching hospitals (NTH) within the United States and their associated outcomes.

## 2 METHOD

Utilizing the National Inpatient Sample Database (NIS), we conducted a retrospective cohort study on hospitalizations with prostate cancer and SCC between 2016 and 2020 across the US. The NIS is a publicly accessible database that covers inpatient hospitalizations across the United States, offering estimates representative of hospital stays on a national scale. This database comprises a stratified sample representing around 20% of discharges from community hospitals throughout the country, equating to estimates for over 35 million hospitalizations each year.^21^ Since the NIS database contains de‐identified information, the study was exempt from review by the institutional review board.

We identified all adult admissions with an underlying diagnosis of prostate cancer and cord compression using appropriate ICD‐10‐CM codes, which are listed in detail in the supplemental material. Admissions were further categorized into teaching and non‐teaching hospitals based on hospital classification, and subdivided based on whether they received surgical intervention, radiation therapy, or no intervention. Baseline variables, including demographic characteristics such as age, primary insurance, race, and comorbidities (identified using the Charlson comorbidity index), as well as outcomes (mortality, disposition, length of stay, and hospitalization cost), were compared between prostate cancer and SCC admissions in teaching and non‐teaching hospitals. Additionally, we conducted comparisons of baseline characteristics and outcomes among admissions that underwent different interventional modalities (i.e., surgery, radiation, or neither).

Descriptive analysis was performed after applying sampling weights, using the methodology provided by the Healthcare Cost and Utilization Project. We used Pearson's chi‐square test to obtain percentages for categorical variables and the epctile (estimation and inference for percentiles) command to estimate weighted medians of continuous variables, including the interquartile range (IQR). The analyses were performed using Stata software, version 15.1 (StataCorp, College Station, TX).

## 3 RESULTS

In this study, we identified 11,380 admissions who had metastatic prostate cancer with spinal cord compression. Among them, 9610 were treated in teaching hospitals (TH), and 1770 in non‐teaching hospitals (NTH). The median age of these patients was 69; there was no significant difference in age between TH and NTH patients (*p*: 0.1173). Regarding the racial distribution, 52.9% of patients were white, 29.9% black, 10.2% Hispanic, and 7.0% belonged to other races. With regards to insurance status, 60.7% had Medicare, 12.6% Medicaid, 20.8% private insurance, and 5.9% were self‐pay. There were no significant differences in insurance coverage between TH and NTH patients (*p*: 0.3185). The Charlson comorbidity index showed no significant differences between the two groups, as shown in Table 1.

**TABLE 1:** Comparing patients' baseline characteristics in teaching vs non‐teaching hospital admitted between 2016 and 2020 across the US who had spinal cord compression in the setting of metastatic prostate cancer.

Baseline characteristics	All admissions (*N* = 11,380)	Teaching hospitals (*N* = 9610)	Non‐teaching hospitals (*N* = 1770)	*p* value
Age on admission in years, median (IQR)	69 (62–77)	69 (62–77)	70 (63–78)	0.1173
Race				0.0011
White	52.9%	51.5%	60.5%	
Black	29.9%	31.2%	22.5%	
Hispanic	10.2%	9.8%	12.7%	
Other^a^	7.0%	7.5%	4.3%	
Primary expected payer				0.3185
Medicare	60.7%	60.0%	64.7%	
Medicaid	12.6%	12.6%	12.7%	
Private Insurance	20.8%	21.5%	17.2%	
Self‐pay & other	5.9%	6.0%	5.4%	
Charlson comorbidity index				0.6605
≤2	0.8%	0.9%	0.6%	
3–4	2.1%	2.0%	2.5%	
>4	97.1%	97.1%	96.9%	

Abbreviation: IQR, Interquartile range.

^a^ Other races: Asian or Pacific Islander, Native American and others.

With regards to the management of spinal cord compression in metastatic prostate cancer patients, 28.2% of TH patients underwent surgery compared to 23.0% of NTH patients. Radiation therapy was given to 12.1% of TH patients and 8.2% of NTH patients, while the majority of patients (59.7% of TH patients, and 68.8% of NTH patients) received no intervention. These differences in treatment choices were statistically significant between TH and NTH (*p*: 0.006). The median length of hospital stay was 7 days for TH versus 6 days for NTH patients (*p*: 0.02). The median cost of hospitalization was $21,922 in TH and $15,141 in NTH (*p*: 0.00003). The all‐cause hospital mortality rate was 4.5% in TH and 7.9% in NTH (*p*: 0.005) as shown in Table 2.

**TABLE 2:** Comparing admissions' outcome between teaching and non‐teaching hospitals across US.

Admissions' Outcome	All admissions (*N* = 11,380)	Teaching hospitals (*N* = 9610)	Non‐teaching hospitals (*N* = 1770)	*p* value
Intervention				0.0064
Surgery	27.4%	28.2%	23.0%	
Radiation	11.5%	12.1%	8.2%	
No intervention	61.2%	59.7%	68.8%	
All‐cause in‐hospital mortality	5.0%	4.5%	7.9%	0.0059
Disposition of Patient				0.002
Routine	23.8%	24.2%	21.6%	
Transfer to another short‐term hospital	6.0%	5.2%	10.8%	
Transfer to a facility^a^	46.8%	47.0%	45.4%	
Home‐health care	23.4%	23.6%	22.2%	
Length of stay in days, median (IQR)	7 (4–12)	7 (4–12)	6 (4–10)	0.025
Total hospital costs, median (IQR)	$20,917 (11,066 ‐ 37,309)	$21,922 (12,061 ‐ 38,609)	$15,141 (8414 – 28,591)	0.0003

Abbreviation: IQR, Interquartile range.

^a^ Facilities include skilled nursing facility, intermediate care.

In teaching hospitals, a higher proportion of black patients received radiation (34.2%) compared to surgery (26.8%) and no intervention (32.7%) (*p*: 0.03), as shown in Table 3. A higher proportion of patients with private insurance underwent surgery (25.1%) compared to radiation (18.8%) and no intervention (20.1%) (*p*: 0.03). The Charlson comorbidity index did not differ significantly among surgery, radiation, and no intervention groups (*p*: 0.16). The median length of stay was 8 days for surgery, 9 days for radiation, and 7 days for no intervention (*p*: 0.00014). The median cost of hospitalization was $36,345 for surgery, $23,215 for radiation, and $15,691 for the no intervention group (*p*: 0.001). The all‐cause hospital mortality rate was 1.9% for surgery, 4.4% for radiation, and 5.7% for no intervention (*p*: 0.0016).

**TABLE 3:** Surgery vs radiation vs no surgery or radiation comparison in teaching hospitals across US.

Baseline characteristics	Surgery (*N* = 2680)	Radiation (*N* = 1145)	No surgery or radiation (*N* = 5675)	*p* value
Age on admission in years, median (IQR)	68 (62–74)	68 (62–78)	70 (62–77)	0.0042
Race				0.039
White	55.2%	50.9%	49.9%	
Black	26.8%	34.2%	32.7%	
Hispanic	11.4%	5.4%	9.7%	
Other^a^	6.6%	9.5%	7.6%	
Primary expected payer				0.0338
Medicare	54.8%	66.8%	61.3%	
Medicaid	12.8%	10.5%	12.9%	
Private Insurance	25.1%	18.8%	20.1%	
Self‐pay & other	7.3%	3.9%	5.7%	
Charlson comorbidity index				0.1672
≤2	0.8%	0.4%	1.1%	
3–4	2.1%	0.0%	2.4%	
> 4	97.2%	99.6%	96.6%	
All‐cause in‐hospital mortality	1.9%	4.4%	5.7%	0.0016
Disposition of patient				<0.001
Routine	19.4%	26.0%	26.3%	
Transfer to another short‐term hospital	3.2%	0.5%	7.0%	
Transfer to a facility^b^	61.0%	47.5%	40.0%	
Home‐health care	16.4%	26.0%	26.7%	
Length of stay in days, median (IQR)	8 (6–12)	9 (6–15)	7 (3–12)	0.0014
Total hospital costs, median (IQR)	$36,345 (24,479–57,182)	$23,215 (14,880–35,685)	$15,691 (8635–29,171)	<0.001

Abbreviation: IQR, Interquartile range.

^a^ Other races: Asian or Pacific Islander, Native American and others.

^b^ Facilities include skilled nursing facility, intermediate care.

In NTH, a higher proportion of patients with private insurance underwent surgery (27%) compared to radiation (6.9%) and no intervention (15.3%) (*p*: 0.02). However, while the Charlson comorbidity index was not significantly different among the surgery, radiation, and no intervention groups (Table 4, *p*: 0.7), all‐cause hospital mortality rate was 4.9% for surgery, 6.9% for radiation, and 9.1% for no intervention (*p*: 0.4). The median length of stay was 8 days for surgery, 7 days for radiation, and 6 days for no intervention (*p*: 0.09). The median cost of hospitalization was $28,449 for surgery, $19,899 for radiation, and $10,735 for the no intervention group (*p*: 0.001).

**TABLE 4:** Surgery vs radiation vs no surgery or radiation comparison in non‐teaching hospitals across US.

Baseline characteristics	Surgery (*N* = 405)	Radiation (*N* = 145)	No surgery or radiation (*N* = 1210)	*p* value
Age on admission in years, median (IQR)	67 (62–75)	70 (63–79)	70 (63–79)	0.1262
Race				0.1746
White	53.9%	64.3%	61.9%	
Black	20.5%	25.0%	23.0%	
Hispanic	21.8%	10.7%	10.0%	
Other^a^	3.9%	0.0%	5.0%	
Primary expected payer				0.0271
Medicare	59.3%	69.0%	66.1%	
Medicaid	12.4%	10.3%	12.8%	
Private Insurance	27.0%	6.9%	15.3%	
Self‐pay & other	1.2%	13.8%	5.8%	
Charlson comorbidity index				0.7669
≤2	0.0%	0.0%	0.8%	
3–4	2.5%	0.0%	2.9%	
> 4	97.5%	100.0%	96.3%	
All‐cause in‐hospital mortality	4.9%	6.9%	9.1%	0.4822
Disposition of Patient				<0.001
Routine	16.9%	25.9%	22.5%	
Transfer to another short‐term hospital	1.3%	3.7%	15.1%	
Transfer to a facility^b^	70.1%	48.2%	36.2%	
Home‐health care	11.7%	22.2%	26.2%	
Length of stay in days, median (IQR)	8 (6–10)	7 (5–16)	6 (3–10)	0.0976
Total hospital costs in US dollars, median (IQR)	$28,449 (22,402–46,740)	$19,899 (14,356–27,900)	$10,735 (7089–21,054)	<0.001

Abbreviation: IQR, Interquartile range.

^a^ Other races: Asian or Pacific Islander, Native American and others.

^b^ Facilities include skilled nursing facility, intermediate care.

## 4 DISCUSSION

Management of spinal cord compression in patients with metastatic prostate cancer remains challenging, with several factors involved in deciding treatment modality or modalities. Despite no differences in median age and Charlson comorbidity scores between the NTH and TH, patients in TH were more likely to receive intervention (either radiation or surgery) compared to NTH (Surgery: 28.2% in TH vs. 23.0% in NTH; *p* < 0.01 & Radiation: 12.1% in TH vs. 8.2% in NTH; *p* < 0.01). Given the body of evidence regarding the management of spinal cord compression, including validated scales such as the Spine Instability Neoplastic Score to evaluate for surgical intervention, the significant differences in management of cord compression in TH vs NTH is of concern.

For spinal metastases in general, there is high‐grade evidence that supports surgical management of bony metastases that result in myelopathy and high‐grade spinal cord compression as measured by the epidural spinal cord compression (ESCC) scale.^15^, ^22^ While medical comorbidities and tumor burden must be considered when considering surgical intervention, as encompassed by the Neurologic, Oncologic, Mechanical, Systemic (NOMS) framework, our patients did not differ significantly by age or Charlson comorbidity scores, as previously mentioned.^23^

Given that the Charlson comorbidity scale is widely validated indicator of contraindications to surgical intervention, the insignificant difference between median age and Charlson comorbidity scores between the two groups suggests that patients from both groups were equally viable surgical candidates.^24^ Furthermore, while over 95% of both cohorts had Charlson comorbidity scores of over 4, prior literature indicates that surgical cord decompression can be performed in patients with Charlson scores >6, particularly in older patient populations, with significant neurologic improvement.^25^ Although elevated Charlson comorbidity scores are associated with increased risk of postoperative complications significant differences in management of patients with similar comorbidity scores is a concerning finding and indicates inconsistency in clinical practice/decision‐making despite well‐established guidelines.^14^

Among the 11,380 patients in our cohort, the median cost of hospitalization was $21,922 in teaching hospitals, vs $15,141 in non‐teaching hospitals, both higher than prior literature and indicative of the rising costs associated with cancer‐related care.^26^ The difference in cost between teaching and non‐teaching hospitals is striking, as the cost of admission in teaching hospital was nearly one standard deviation above prior documented costs of admission for metastatic cancer.^27^ The discrepancy in cost can in part be explained by differences in management, as patients in teaching hospitals were significantly more likely to receive “costly” interventions like surgery or radiation. Furthermore, patients at academic centers tend to accrue higher costs for cancer‐related care.^28^

There are concerning differences in mortality due to variation in management between TH and NTH. In part, this mortality difference can in part be explained by the increased rates of intervention (surgery and radiation) in the TH vs NTH cohort. While prior literature has found differences in medical comorbidities at TH vs NTH, there was no significant difference in age or comorbidity scores between the two cohorts.^29^ While individual factors may influence the decision to pursue inpatient interventions such as surgical decompression and radiation, the significant mortality difference between NTH and TH warrants further investigation. Among patients who did receive an intervention at teaching hospitals, there were differences in mortality by treatment modality. Despite no differences in comorbidities between surgery and radiation, all‐cause mortality was higher for those who received radiation than for surgery (4.4% vs. 1.9%; *p* < 0.05). While comorbidities play a role in all‐cause mortality, other factors could potentially explain the mortality difference between surgical vs non‐surgical candidates, including tumor burden and performance status.^30^

Both socioeconomic and demographic variables were associated with differences in management of patients with prostate cancer. Common to both TH and NTH, a higher proportion of patients with private insurance underwent surgery compared to radiation therapy, consistent with prior literature.^16^ Given that surgery was associated with improved mortality compared to radiation therapy, it follows that patient with non‐private insurance may not receive the same quality of interventions as patients with private insurance. This would be consistent with prior findings that uninsured patients and patients with Medicare or other forms of non‐private insurance are less likely to receive surgical care, wait significantly longer for surgical interventions, and receive lower rates of guideline‐based cancer therapy.^31^, ^32^, ^33^ While the relationship between insurance status and prostate cancer‐related mortality is controversial, our findings that patients with non‐private insurance receive lower rates of surgical interventions (compared to private insurance) adds to existing literature suggesting that insurance status correlates with quality of care in patients with prostate cancer.^34^, ^35^

Black patients were more likely to receive radiation than surgery in TH (34.2% vs. 26.8%; *p* < 0.05), compared to non‐black counterparts. Similarly, several studies found that Black men were more likely to receive radiation than surgery as first treatment.^17^, ^18^, ^19^ While all‐cause mortality by race was not directly measured in our study, across all races we found that receiving radiation as opposed to surgical intervention was associated with higher risk of all‐cause mortality. It follows that by receiving radiation instead of surgery, patients may have had a higher risk of death and other adverse outcomes compared to their non‐black counterparts. This would be consistent with a multivariate risk model developed with 25 years of clinical data, which found that Black men with prostate cancer men experience higher rates of mortality than non‐Hispanic white men.^19^ Furthermore, at the intersection of these two groups, Black patients with Medicare/Medicaid were found to be less likely to receive guideline‐based therapy for cancer care, highlighting the interplay of race and insurance in poor care outcomes.^33^ Regardless of hospital setting, the role of insurance and race in the care of prostate cancer warrants further investigation.

## 5 CONCLUSION

Our study demonstrates that management of spinal cord compression in US patients with metastatic prostate cancer and all‐cause mortality differ by hospital setting. Moreover, differences in management and clinical outcomes by race and insurance status highlight disparities in prostate cancer care on a national level. Future studies are needed to investigate these discrepancies and work toward standardized management for spinal cord compression, regardless of a patient's hospital setting, race, or insurance status.

### 5.1 Limitations of study

While we utilized a large nationwide database for this study, it is important to interpret the results with caution. The database relies on ICD‐10 codes for disease and procedure identification, which introduces a risk of misclassification due to inaccurate coding. Additionally, distinguishing between comorbidities and complications occurring during hospitalization may not be precise. The retrospective studies are prone to selection bias and this descriptive study does not allow for firm conclusions. The findings should be validated in future studies with more detailed analysis to identify the association between teaching status and modality of intervention. Lastly, these results from the US might not be generalizable to other countries, where standard treatment approaches may differ due to varying preferences and healthcare practices.

## AUTHOR CONTRIBUTIONS

**Omid Yazdanpanah:** Conceptualization (equal); data curation (supporting); formal analysis (supporting); investigation (equal); methodology (equal); validation (equal); writing – original draft (equal); writing – review and editing (equal). **Aditya Mahadevan:** Investigation (equal); methodology (equal); validation (equal); writing – original draft (equal); writing – review and editing (equal). **Aditi Sharma:** Data curation (lead); formal analysis (lead); investigation (equal); methodology (equal); validation (equal); writing – original draft (equal); writing – review and editing (equal). **David J. Benjamin:** Investigation (equal); methodology (equal); supervision (equal); validation (equal); writing – original draft (equal); writing – review and editing (equal). **Arash Rezazadeh Kalebasty:** Conceptualization (equal); data curation (supporting); formal analysis (supporting); investigation (equal); methodology (equal); supervision (lead); validation (equal); writing – original draft (equal); writing – review and editing (equal).

## CONFLICT OF INTEREST STATEMENT

OY, AM, and AS have no conflicts of interest to disclose. DJB has received consulting fees from Seagen. ARK declares stock and other ownership interests: ECOM Medical; consulting or advisory role: Exelixis, AstraZeneca, Bayer, Pfizer, Novartis, Genentech, Bristol Myers Squibb, EMD Serono, Immunomedics and Gilead Sciences; speakers' bureau: Janssen, Astellas Medivation, Pfizer, Novartis, Sanofi, Genentech/Roche, Eisai, AstraZeneca, Bristol Myers Squibb, Amgen, Exelixis, EMD Serono, Merck, Seattle Genetics/Astellas, Myovant Sciences, Gilead Sciences and AVEO; research funding: Genentech, Exelixis, Janssen, AstraZeneca, Bayer, Bristol Myers Squibb, Eisai, Macrogenics, Astellas Pharma, Beyond Spring Pharmaceuticals, BioClin Therapeutics, Clovis Oncology, Bavarian Nordic, Seattle Genetics, Immunomedics and Epizyme; and travel, accommodations and/or expenses: Genentech, Prometheus, Astellas Medivation, Janssen, Eisai, Bayer, Pfizer, Novartis, Exelixis and AstraZeneca.

## Data Availability

The data that support the findings of this study are openly available in HCUP‐US NIS Overview. Agency for Healthcare Research and Quality at https://hcup‐us.ahrq.gov/nisoverview.jsp.^21^
